# Molecular pathways involved in the control of contractile and metabolic properties of skeletal muscle fibers as potential therapeutic targets for Duchenne muscular dystrophy

**DOI:** 10.3389/fphys.2024.1496870

**Published:** 2024-12-09

**Authors:** Agnese Bonato, Giada Raparelli, Maurizia Caruso

**Affiliations:** Institute of Biochemistry and Cell Biology, National Research Council (CNR), Monterotondo (RM), Italy

**Keywords:** duchenne muscular dystrophy, skeletal muscle fiber types, skeletal muscle metabolism, phenotypic modifiers, therapeutic strategies

## Abstract

Duchenne muscular dystrophy (DMD) is caused by mutations in the gene encoding dystrophin, a subsarcolemmal protein whose absence results in increased susceptibility of the muscle fiber membrane to contraction-induced injury. This results in increased calcium influx, oxidative stress, and mitochondrial dysfunction, leading to chronic inflammation, myofiber degeneration, and reduced muscle regenerative capacity. Fast glycolytic muscle fibers have been shown to be more vulnerable to mechanical stress than slow oxidative fibers in both DMD patients and DMD mouse models. Therefore, remodeling skeletal muscle toward a slower, more oxidative phenotype may represent a relevant therapeutic approach to protect dystrophic muscles from deterioration and improve the effectiveness of gene and cell-based therapies. The resistance of slow, oxidative myofibers to DMD pathology is attributed, in part, to their higher expression of Utrophin; there are, however, other characteristics of slow, oxidative fibers that might contribute to their enhanced resistance to injury, including reduced contractile speed, resistance to fatigue, increased capillary density, higher mitochondrial activity, decreased cellular energy requirements. This review focuses on signaling pathways and regulatory factors whose genetic or pharmacologic modulation has been shown to ameliorate the dystrophic pathology in preclinical models of DMD while promoting skeletal muscle fiber transition towards a slower more oxidative phenotype.

## 1 Introduction

Duchenne Muscular Dystrophy (DMD) is a severe and incurable X-linked disorder characterized by progressive loss of skeletal muscle mass and function and cardiomyopathy (Duan et al., 2021). DMD is caused by loss of function mutations in the gene encoding dystrophin, a structural protein located at the intracellular surface of the sarcolemma that links the actin cytoskeleton of skeletal myofibers to the extracellular matrix through its interaction with transmembrane components of the multifunctional dystrophin-associated protein complex (DAPC). Together, dystrophin and the DAPC maintain sarcolemmal stability thus preventing damage from mechanical stresses (Ervasti, 2007). In DMD*,* the absence of functional dystrophin, and subsequent disassembly of the DAPC, results in high susceptibility of the sarcolemma to contraction-induced injury causing an increase of calcium (Ca^2+^) influx into dystrophic fibers, protease activation and free radical formation as well as loss of neuronal nitric oxide synthase (nNOS) membrane localization and consequent impairment of nitric oxide (NO) signaling (Allen et al., 2016). The chronic sarcolemmal damage leads to myofiber necrosis, persistent inflammation and compensatory regeneration mediated by resident stem cells (satellite cells); however, in later phases of the disease the tissue regenerative capacity becomes exhausted, owing to intrinsic dysfunction of dystrophin-deficient satellite cells and compromised niche microenvironment, and dead myofibers are ultimately replaced by fibrotic and fatty tissue (Dumont et al., 2015; Cappellari et al., 2020). These morphological changes are associated with mitochondrial dysfunction, a decrease in oxidative capacity of the muscle, impaired ATP production, and numerous metabolic abnormalities (Heydemann, 2018).

Currently there is no cure for DMD and its symptomology is treated with glucocorticoids, which delay disease progression and prolong ambulation by exerting an anti-inflammatory action (McDonald et al., 2018). However, their long-term use leads to adverse side effects, including muscle atrophy and metabolic dysfunctions such as inhibition of insulin production, hyperglycemia, insulin resistance, fatty liver, obesity. Multiple approaches are being pursued to attenuate these side effects, such as intermittent dosing and development of novel glucocorticoid derivatives (Quattrocelli et al., 2021).

There are, several therapeutic interventions targeting DMD genetic defects that have shown promise in preclinical and clinical trials, including restoration of dystrophin production through viral delivery of microdystrophin genes, exon skipping drugs, stop codon readthrough, gene editing, and cell-based therapies. The current status of genetically based DMD therapies and their advantages and disadvantages have been discussed in detail in several recent reviews (Roberts et al., 2023; Bez Batti Angulski et al., 2023; Chang et al., 2023; Deng et al., 2022; Markati et al., 2022). Furthermore, therapeutic strategies targeting pathological mechanisms downstream of dystrophin deficiency are also being developed. These include anti-inflammatory, antioxidant and anti-fibrotic treatments, and treatments targeting impaired Ca^2+^ and NO signaling, mitochondrial dysfunction, myofiber necrosis, impaired regenerative capacity (recently reviewed by Markati et al., 2022; Angelini et al., 2022; Sandonà et al., 2023). These therapeutic approaches apply to all DMD genotypes and are strongly envisaged because by protecting from degeneration and preserving muscle function, they are expected to increase quality of life, prolong survival, and improve the effectiveness of pharmacologic, genetic and cell-based therapies. Within this context, a potential therapeutic strategy aiming to preserve the musculature in DMD involves the remodeling of dystrophic muscle towards a slower, more oxidative phenotype, which has been shown to be more resistant to the dystrophic pathology than the faster more glycolytic phenotype (Webster et al., 1988; Ljubicic et al., 2014a).

The purpose of this review is to provide an overview and discussion of preclinical and clinical evidence suggesting that DMD progression may be countered by promoting a slower, more oxidative phenotype of skeletal muscle fibers. We briefly describe the contractile and metabolic characteristics of the various muscle fiber types that could explain their differential susceptibility to contraction-induced damage. Then, we focus on molecular pathways and effectors involved in phenotypic remodeling of skeletal muscle, whose genetic or pharmacological modulation has been shown to benefit the dystrophic pathology.

## 2 Skeletal muscle fiber types

Skeletal muscle groups consist of heterogeneous and specialized myofibers that differ in their speed of contraction (slow or fast) and metabolism (oxidative or glycolytic). Myofibers are broadlly classified into slow-twitch (type I) and fast-twitch (type II) based on contractile performance and expression of slow/type I or fast/type II myosin heavy chain (MyHC) isoforms. Fast-twitch fibers are further classified into three subtypes (type IIa, type IIx and type IIb), based on the expression of specific isoforms of fast MyHC. Slow/typeI and fast/typeIIa fibers primarily use oxidative metabolism, display high mitochondrial content and capillary density, consume less ATP during contraction, generate less reactive oxygen species (ROS), and are resistant to fatigue. Fast/IIb fibers primarily rely upon glycolytic metabolism and display considerable strength and contraction speed, but only for short bursts of activity, and are therefore quickly fatigable. Fast/IIx fibers utilize a mixed glycolytic/oxidative metabolism and their resistance to fatigue is intermediate between that of IIb and IIa fibers (reviewed by Schiaffino and Reggiani, 2011; Blaauw et al., 2013). A similar fiber type profile has been observed in different mammalian species; however, in human muscles MyHCIIb is undetectable and fibers typed as IIb by histochemical staining express in fact the MyHCIIx isoform (Smerdu et al., 1994). Muscle fiber type diversification is established during embryonic development and reflects different patterns of gene expression, but the adult fiber type profile emerges postnatally through the combined action of intrinsic genetic programs and extrinsic factors, such as hormonal and neural cues (Schiaffino and Reggiani, 2011). In adult muscle, specialized myofibers remain plastic and can remodel their metabolic and contractile profile in response to environmental demands and new pathophysiological conditions by activating signaling pathways to reprogram gene expression (Blaauw et al., 2013).

## 3 Differential susceptibility of fast/glycolytic and slow/oxidative fibers to the dystrophic pathology

Impaired energy metabolism and mitochondrial dysfunction are well-known pathological features of DMD (Timpani et al., 2015). Dystrophin-deficient muscles have reduced ATP concentrations, which is a consequence of not only an increased demand for ATP that is required for Ca^2+^ buffering and muscle regeneration but also an impaired ATP production capacity of dystrophic mitochondria (Kuznetsov et al., 1998; Rybalka et al., 2014; Onopiuk et al., 2009). In fact, a plethora of structural and functional abnormalities have been documented in dystrophic mitochondria, including swollen morphology and changes in cristae shape and density, impaired oxidative phosphorylation, decreased activity of complex I of the, electron transport chain, and elevated mitochondrial ROS emission (Timpani et al., 2015; Moore et al., 2020; Kuznetsov et al., 1998; Rybalka et al., 2014; Hughes et al., 2019).

Importantly, a comprehensive analysis of multiple expression profiling datasets revealed that genes downregulated in DMD muscles are commonly associated with energy metabolism and muscle contraction and that almost all regulators of these genes correspond to a single common pathway important for fast/glycolytic-to-slow/oxidative fiber type transition (Kotelnikova et al., 2012). It has been known for many years that fast glycolytic muscle fibers are preferentially affected both in DMD patients and DMD mouse models compared to their slower, oxidative counterparts (Webster et al., 1988; Pedemonte et al., 1999; Moens et al., 1993). Accordingly, a fast-to-slow fiber type shift is seen in dystrophic muscles, which may reflect the selective degeneration of faster/glycolityc fibers as well as a compensatory transition to slower/oxidative fibers that are more resistant to severe dystrophic pathology (Petrof et al., 1993; Baker et al., 2006; Rafael et al., 2000), probably due to a combination of their properties, including lower ATP consumption during contraction, decreased energy requirements, and higher oxidative and mitochondrial capacity (Schiaffino and Reggiani, 2011).

Notably, extraocular muscles (EOMs) exhibit remarkable resistance to DMD pathology despite being among the fastest skeletal muscles (Titova et al., 2024). At variance with limb muscles, EOMs combine a very high speed of contraction and a high resistance to fatigue. In addition to nearly all known MyHC isoforms, they express an EOM-specific MyHC isoform and retain expression of embryonic isoforms. Furthermore, many EOM fibers express multiple MyHC isoforms (Schiaffino and Reggiani, 2011). Expression profiling revealed EOMs predominantly utilize aerobic carbohydrate metabolism, rely on blood glucose levels as an energy source, and express increased levels of genes required for efficient, fatigue-resistant oxidative metabolism compared to tibialis anterior muscles (Fischer et al., 2002). These peculiar metabolic properties might contribute to the sparing of EOMs in DMD.

### 3.1 Fast fibers

The role of fast skeletal muscle contraction in dystrophic muscle degeneration and disease progression has been recently assessed in animal models of DMD by using a small-molecule selective inhibitor of the ATPase activity of fast myosin, EDG-5506 (Russell et al., 2023). It was shown that even small amounts of fast myosin inhibition in the *mdx* mouse model of DMD resulted in almost complete protection against skeletal muscle membrane injury and force loss during eccentric contraction, without detrimental effects on strength and coordination *in vivo*. Furthermore, 3-weeks exposure of pre-weaning *mdx* mice to EDG-5506 decreased central nucleation and regenerating fibers in soleus muscles, whereas a longer-term treatment starting at 6–7 weeks of age was able to decrease fibrosis not only in the diaphragm muscle of *mdx* mice but also in skeletal muscles and heart of DBA/2J *mdx* mice, a more fibrotic model of DMD. Finally, 14-day oral treatment of dystrophic golden retriever dogs with EDG-5506 was shown to decrease plasma creatine kinase (CK) levels, to increase habitual activity and to reverse proteomic signatures associated with the disease (Russell et al., 2023). Therefore, inhibiting the ability of fast myosin to develop force represents a promising treatment strategy to protect injury-susceptible dystrophic fast skeletal muscle. Clinical trials of EDG-5506 in both patients with DMD and those with Becker muscular dystrophy (BMD) are currently ongoing (Clinical Trial identifiers: NCT05540860, NCT06100887, NCT05160415, NTC04585464, NCT05291091, NCT06066580).

### 3.2 Slow, oxidative fibers

As mentioned above, a combination of several characteristics of slow, oxidative fibers might contribute to their enhanced resistance to DMD, including sustained Ca^2+^ influx, reduced contractile speed, resistance to fatigue, increased capillary density, higher mitochondrial activity, decreased cellular energy requirements (Schiaffino and Reggiani, 2011; Blaauw et al., 2013). Furthermore, slow fibers express increased levels of the dystrophin autosomal paralogue utrophin, which shares a high degree of structural and functional similarity with dystrophin and associates with many components of the DAPC (Matsumura et al., 1992; Winder et al., 1995). Utrophin is normally expressed throughout the sarcolemma in fetal and regenerating muscle but is downregulated and replaced by dystrophin after birth. In mature skeletal muscle, utrophin is confined to the neuromuscular and myotendinous junctions whereas dystrophin is expressed along the entire sarcolemma of myofibers (Blake et al., 1996). However, slower, oxidative fibers display a natural increase of utrophin in extrasynaptic regions as compared with fast glycolytic fibers, and such increase appears to involve the contribution of post-transcriptional mechanisms as well as upregulation of the utrophin A promoter mediated by calcineurin, a calcium/calmodulin-regulated protein phosphatase known to promote the slow oxidative myofiber program by acting via the nuclear factor of activated T cells (NFAT) and myocyte enhancer factor 2 (MEF2) transcription factors (Gramolini et al., 2001; Chakkalakal et al., 2003; Chin et al., 1998).

Utrophin A is upregulated also in dystrophic muscle, where it shows an expression pattern extending outside the neuromuscular junction (NMJ) and can associate with DAPC components, suggesting that utrophin can functionally compensate for the lack of dystrophin and that its overexpression in dystrophic muscle might protect against muscle damage (Karpati et al., 1993; Weir et al., 2002; Kleopa et al., 2006). Accordingly, dystrophin/utrophin double knockout mice display a much more severe phenotype when compared to dystrophin deficient mice (Deconinck A. E et al., 1997). Furthermore, overexpression of truncated or full-length utrophin through transgenic and gene transfer technologies has been shown to restore sarcolemmal expression of DAPC members and ameliorate the dystrophic pathology in *mdx* mice and dystrophic dogs (Tinsley et al., 1996; Deconinck N et al., 1997; Tinsley et al., 1998; Cerletti et al., 2003). However, activation of an inducible utrophin transgene in *mdx* mice 30 days after birth caused more modest improvements, suggesting that the age at which utrophin therapy is initiated could be an important factor (Squire et al., 2002). In addition, it has been shown that stimulation of the calcineurin/NFAT pathway increases utrophin expression and attenuates the dystrophic pathology in *mdx* mice. In fact, overexpression of a constitutively active form of calcineurin in skeletal muscle of *mdx* mice promoted a shift in fiber type towards a slower phenotype and increased the levels of utrophin and DAPC components at the sarcolemma. These changes were associated with improved dystrophic muscle histopathology and function (Chakkalakal et al., 2004; Stupka et al., 2006). Conversely, targeted inhibition of calcium/calmodulin signaling in *mdx* mice caused a shift to a faster more glycolytic myofiber type, decreased expression of utrophin and worsened pathology (Chakkalakal et al., 2006).

In normal skeletal muscle, the neuronal isoform of nitric oxide synthase (nNOS) participates to the DAPC through interactions with syntrophin and dystrophin (Lai et al., 2009). In dystrophic muscle, disruption of the DAPC leads to displacement from the sarcolemma and decreased expression of nNOS, and consequently to diminished generation of NO, a critical signaling molecule involved in many key processes, including the maintenance of adequate blood flow to skeletal muscle during contractile activity (Rando, 2001). In particular, it has been demonstrated that delocalization of nNOS in dystrophin-deficient mouse and human muscles impairs protective compensatory vasodilation during muscle contraction leading to ischemia and necrosis (Thomas et al., 1998; Sander et al., 2000). It is important to note that utrophin, unlike dystrophin, lacks the capacity to anchor nNOS to the sarcolemma, and this may explain the susceptibility to chronic exercise-associated injury observed in muscles of *mdx* mice overexpressing transgenic utrophin (Li et al., 2010). There is evidence that increasing endogenous NO production through administration of L-arginine, the substrate for NOS, promotes utrophin protein expression at the sarcolemma and ameliorates the dystrophic pathology in adult *mdx* mice (Chaubourt et al., 1999; Barton et al., 2005; Voisin et al., 2005). Specifically, L-arginine treatment reduced myofiber necrosis, decreased the susceptibility to contraction-induced damage and improved the contractile properties of isolated extensor digitorum longus (EDL) (Barton et al., 2005) and diaphragm muscles (Voisin et al., 2005). Another study demonstrated that L-arginine supplementation decreased inflammation and enhanced regeneration in the diaphragm of *mdx* mice by downregulating the activity of the pro-inflammatory NF-kB transcription factor. Moreover, L-arginine decreased the activity of metalloproteases, which are activated by NF-kB, thus reducing cleavage of β-dystroglycan and promoting stabilization and membrane localization of utrophin (Hnia et al., 2008). In contrast, early administration of L-arginine in neonatal *mdx* mice did not result in utrophin upregulation, as previously reported in adult *mdx* mice, although the treatment improved muscle histopathology and resistance to eccentric contractions (Dudley et al., 2021). Therefore, the benefits to dystrophic muscle elicited by L-arginine-stimulated increase of NO production cannot be attributed solely to utrophin upregulation. This is consistent with the observation that expression of a nNOS transgene in *mdx* muscle reduced the extent of the dystrophic pathology by decreasing inflammation and muscle membrane damage without increasing utrophin levels (Wehling et al., 2001; Tidball and Wehling-Henricks, 2004).

Although utrophin may not be able to fully compensate for the absence of dystrophin, utrophin upregulation represents a promising non–toxic (Fisher et al., 2001) and non-immunogenic therapeutic strategy (Song et al., 2019) that would be applicable to all patients, regardless of the specific DMD genetic defect. Several therapeutic approaches designed to increase utrophin expression in dystrophic muscle have been advanced, including *i)* adeno-associated virus (AAV)-mediated delivery of micro-utrophin genes, *ii)* upregulation of endogenous utrophin levels through activation of the utrophin A promoter with orally available drugs, artificial zinc finger transcription factors, catalytically inactive dCas9 fused to a transcriptional activation domain, *iii)* strategies aimed at relieving post-transcriptional repression of utrophin mRNA (recently reviewed by Roberts et al., 2023; Szwec et al., 2024).

## 4 Signal pathways and downstream effectors involved in skeletal muscle phenotypic plasticity as potential therapeutic targets for DMD

Given the resistance to the dystrophic pathology and the higher content of utrophin A observed in slow, more oxidative muscle, it was proposed that promoting the slow-oxidative muscle fiber program might represent a strategy to protect against DMD (Ljubicic et al., 2014a). 

In the past 15 years, a relevant number of studies focused on cellular pathways and downstream effectors that regulate fast-to-slow fiber type transition. In addition to calcineurin, these studies identified several factors acting as activators or repressors of the slow/oxidative myogenic program and verified the therapeutic potential of their stimulation or repression in pre-clinical models of DMD.

### 4.1 Activators of the slow/oxidative myogenic program

#### 4.1.1 PGC1α

Peroxisome proliferator-activated receptor (PPAR) γ coactivator 1α (PGC1α), originally identified as a regulator of adaptive thermogenesis (Puigserver et al., 1998), is a potent transcriptional co-activator that plays crucial roles in the regulation of whole-body energy homeostasis and metabolism, both in health and disease (Handschin and Spiegelman, 2006; Chan and Arany, 2014). Specifically, PGC1α regulates the expression of genes involved in mitochondrial biogenesis and oxidative function by coactivating nuclear respiratory factors (NRF)-1 and 2, estrogen related receptor α (Errα), and PPARα (Wu et al., 1999; Mootha et al., 2004; Schreiber et al., 2004; Vega et al., 2000).

In skeletal muscle tissue, PGC1α has been shown to be expressed preferentially in muscles rich in slow fibers, characterized by high mitochondrial content and oxidative rate, and to drive a fast to slow fiber type conversion when transgenically expressed under control of a muscle-specific promoter. Indeed, muscles from PGC1α-expressing mice displayed increased expression of mRNA for mitochondrial genes involved in electron transport, increased levels of slow-fiber specific myofibrillar proteins and greater resistance to fatigue (Lin et al., 2002). PGC1α muscle-specific transgenic animals also displayed enhanced transcription of a broad NMJ gene program, including utrophin (Handschin et al., 2007a). Conversely, PGC1α muscle-specific knockout mice exhibited a shift from slower/oxidative toward glycolytic muscle fibers and decreased endurance capacity, as well as reduced expression of mitochondrial and NMJ genes (Handschin et al., 2007b). Collectively, these studies demonstrate that PGC1α is a key regulator of the slow/oxidative myogenic program. Mechanistically, PGC1α appears to mediate transcription of oxidative, slow fiber-specific genes by co-activating MEF2 transcription factors (Lin et al., 2002). Notably, it has also been reported that the PGC1α promoter is a direct target of calcium-signaling factors and that PGC1α positively regulates its own transcription by co-operating with MEF2 transcription factors (Handschin et al., 2003). These findings highlight the existence of a positive feedback loop between PGC1α and MEF2 in the coordinated regulation of metabolic and contractile properties of slow fibers. Furthermore, PGC1α has been shown to promote fast-to-slow fiber-type switch by inducing the expression of hypoxia-inducible factor 2α (Hif2α), a key transcriptional regulator of muscle fiber type determination (Rasbach et al., 2010).

Several studies have revealed beneficial effects of PGC1α overexpression in dystrophic muscle. Indeed, skeletal muscle-specific expression of a PGC1α transgene in *mdx* mice was shown to improve muscle histopathology and exercise performance, decrease plasma CK levels, and increase utrophin mRNA and protein levels as well as expression of genes involved in the NMJ program and oxidative phosphorylation (Handschin et al., 2007a). Importantly, skeletal muscle-specific PGC1α transgene expression was able to ameliorate muscle damage also in mice lacking both dystrophin and utrophin, indicating that PGC1α can protect against muscular dystrophy independently of utrophin (Chan et al., 2014).

PGC1α has been shown to improve muscular dystrophy even if activated post-natally. In fact, AAV-mediated PGC1α gene transfer in muscles of neonatal *mdx* mice resulted in a shift toward slower fiber types, increased expression of utrophin and mitochondrial proteins, and greater resistance to contraction-induced damage and fatigue, as observed at 6 weeks of age (Selsby et al., 2012). Furthermore, by using an *mdx* mouse model expressing an inducible skeletal muscle-specific PGC1α transgene, Chan et al. (2014) showed that induction of PGC1α for 4 weeks starting at weaning was able to reduce myofiber damage, centrally nucleated myofibers, and serum CK levels. Moreover, dystrophic muscles of 6-weeks-old *mdx* mice 3 weeks following AAV-mediated PGC1α gene transfer were shown to display less muscle damage, improved resistance to contraction-induced injury and fatigue and increased levels of utrophin protein throughout the sarcolemma. PGC1α treated muscles also showed increased expression of genes encoding DAPC members and genes related to oxidative metabolism, muscle repair, lysosomal biogenesis and autophagy (Hollinger et al., 2013; Spaulding et al., 2020a). However, a few genes related to inflammation and apoptosis were also found induced in muscles overexpressing PGC1α compared with control muscles (Hollinger et al., 2013). Another study has demonstrated that short term plasmid-mediated PGC1α gene transfer in muscles of 6-week-old *mdx* mice (i.e., during the early post-necrotic phase) was able to rescue various mitochondrial functional abnormalities (Godin et al., 2012). The effects of PGC1α gene delivery were also examined in dystrophic muscles of *mdx* mice aged 12 months. Three months following AAV-mediated gene transfer, the soleus muscle displayed enhanced specific tension and resistance to fatigue, but histopathology was not improved and no relevant differences in gene expression were appreciated between PGC1α-treated and untreated *mdx* limbs (Hollinger and Selsby, 2015). Collectively, these studies indicate that PGC1α pathway activation strategies may prevent disease onset and attenuate pathology at early stages of disease but are less effective when initiated in the context of advanced disease.

As a master regulator of mitochondrial biogenesis and function, PGC1α is tightly modulated in response to energy demands and changing conditions through the regulation of its expression and through the regulation of its activity by several post-translational modifications, such as phosphorylation, acetylation, ubiquitination, methylation, among others (Fernandez-Marcos and Auverx, 2011). Specifically, PGC1α activity is targeted by two crucial sensors of the metabolic status of the cell and the availability of substrates: the adenosine monophosphate-activated protein kinase (AMPK) and the silent mating type information regulator 2 homologue 1 (SIRT1) deacetylase (Canto and Auverx, 2009).

#### 4.1.2 AMPK

AMPK is a serine-threonine kinase that is activated in low energy states by the increase in the intracellular AMP/ATP ratio. AMPK exists as a heterotrimeric complex, composed of a catalytic α subunit, a scaffolding β subunit, and a regulatory γ subunit. In mammals, each of these subunits is encoded by multiple isoforms (α_1_, α_2_, β_1_, β_2_, γ_1_, γ_2_, γ_3_) that are expressed in varying proportions in different tissues, enabling the formation of 12 αβγ heterotrimer combinations (Ross et al., 2016). AMPK is allosterically activated by AMP binding to the γ subunit, which enhances the phosphorylation of a key residue within the catalytic α subunit (Thr^172^) by liver kinase B1 (LKB1) resulting in full AMPK activation. AMPK is also activated by a rise in intracellular Ca^2+^ concentration through phosphorylation of α-Thr^172^ by CAMKK2 (calcium/calmodulin-dependent kinase 2). Once activated, AMPK switches on catabolic pathways that generate ATP, while simultaneously repressing ATP-consuming anabolic pathways (Hardie et al., 2016; Garcia and Shaw, 2017). These actions of AMPK not only take place through modulation of the activity of key metabolic enzymes via direct phosphorylation, but also through the activation of transcriptional regulators that control mitochondrial gene expression and oxidative metabolism (Canto and Auwerx, 2010). Among these, AMPK has been shown to activate through direct phosphorylation the master regulator of mitochondrial biogenesis PGC1α, and this phosphorylation by AMPK also increases PGC1α-dependent induction of its own gene promoter (Jäger et al., 2007). Furthermore, AMPK phosphorylates different members of the forkhead box O (FOXO) family of transcription factors that regulate oxidative stress resistance, energy metabolism and induction of autophagy (Greer et al., 2007).

In skeletal muscle, AMPK mediates adaptations to energy stresses, such as contractile activity during exercise and low nutrient availability, by modulating multiple pathways involved in the control of muscle plasticity, glucose uptake and fatty acid oxidation, mitochondrial function*,* protein metabolism, autophagy (reviewed by Kjobsted et al., 2018).

Muscle specific knockout and transgenic models indicate important roles for AMPK in the regulation of mitochondrial function and in promoting a slower more oxidative muscle fiber type under conditions of energetic stress, such as exercise. Muscle-specific transgenic expression of an AMPKα2 inactive subunit significantly reduced the shift towards more oxidative fibers (type IIb to IIa/x) induced by chronic exercise. Conversely, skeletal muscle from mice expressing an AMPK activating mutation in the γ_1_ subunit showed a higher proportion of IIa/x fibers in sedentary conditions but no further increase after training (Röckl et al., 2007). Furthermore, knockout of the AMPK upstream kinase LKB1 in skeletal and cardiac muscle resulted in impaired exercise capacity, reduced levels of mitochondrial oxidative proteins, increased fatigue and a shift toward more glycolytic fibers (type IIa/x to IIb) in skeletal muscle (Thomson et al., 2007; Thomson et al., 2010). There are however some ambiguities about the role of AMPK in muscle fiber type specification, as overexpression of a gain-of-function mutant form of the AMPKγ_3_ subunit increased mitochondrial biogenesis and metabolic adaptation in glycolytic skeletal muscle but not fiber type reprogramming (Garcia-Roves et al., 2008). Furthermore, muscle-specific AMPKβ1/β2 knockout mice displayed impaired capacity for treadmill running in association with reduced mitochondrial content in skeletal muscle but not a fiber-type switch (O’Neill et al., 2011). Similarly, mice lacking both AMPKα1 and AMPKα2 in skeletal muscle displayed reductions in exercise tolerance and basal mitochondrial function while a compensatory increase in the proportion of MHC type I fibers, with no significant change in the proportion of oxidative type IIa fibers, was reported in muscles of these mice at rest (Lantier et al., 2014). However, these studies did not test muscle fiber type adaptation in AMPK α1/α2 and AMPKβ1/β2 deficient mice after exercise training.

Several studies consistently demonstrated that chronic activation of AMPK in *mdx* mice attenuates the dystrophic pathology while promoting slower, more oxidative muscle characteristics. Indeed, activation of AMPK in 5 to 7-week-old *mdx* mice by administration of the AMP mimetic 5-aminoimidazole-4-carboxamide-1β-D-ribofuranoside (AICAR) has been shown to induce an increase in oxidative fibers and resistance to contraction-induced damage in fast glycolytic muscles, in concomitance with enhanced expression of utrophin A in extrasynaptic regions, increased sarcolemmal integrity, and increased expression of PGC1α protein levels. Notably, such signs of improved pathology were not observed in the slow soleus muscle, already highly oxidative (Ljubicic et al., 2011). Interestingly, previous chronic AICAR treatment of *mdx* mice attenuated the adaptive response to a subsequent physiological stimulus known to induce AMPK signaling, such as acute treadmill exercise (Ljubicic et al., 2012). Another study showed that a 4-week AICAR treatment given to 12-week-old *mdx* mice increased the number of type I fibers in soleus muscle and the oxidative capacity of both EDL and soleus muscles as well as the levels of utrophin protein. These changes were accompanied by significant improvements in disease phenotype, as indicated by a decrease in the number of damaged and regenerating myofibers, decreased fibrosis in diaphragm and increased EDL twitch force parameters (Jahnke et al., 2012). A further study from Jasmin’s laboratory addressed the issue whether the beneficial effects of AICAR-mediated AMPK activation observed in *mdx* mice were solely due to increased expression of utrophin A by examining *mdx* mice also deficient in utrophin (double knockout mice, dKO). AICAR treatment increased the oxidative capacity and induced a shift toward a slower fiber type in the EDL muscle of both *mdx* and dKO mice. However, AICAR enhanced sarcolemmal integrity and expression of DAPC components, and improved muscle function in *mdx* but not dKO mice. Therefore, utrophin A appears to be dispensable for fiber-type shifting, but essential in mediating the functional benefits associated with the slower, more oxidative muscle phenotype induced by AMPK activation in dystrophic animals (Al-Rewashdy et al., 2015).

It has been demonstrated that the autophagic process is severely impaired in muscles of *mdx* mice and DMD patients and that such defect contributes to the pathogenesis of disease (De Palma et al., 2012). Interestingly, reactivation of autophagy in *mdx* mice through long-term exposure to a low protein diet was able to improve the dystrophic phenotype, suggesting autophagy as a therapeutic target in DMD (De Palma et al., 2012). AMPK activation through chronic treatment of *mdx* mice with AICAR has been reported to potently trigger the autophagy pathway, improve mitochondrial morphology and function and increase slow type I fibers in the diaphragm muscle, with associated improvements in histopathology and force-generating capacity. However, diaphragms of AICAR treated *mdx* mice did not show utrophin protein upregulation (Pauly et al., 2012).

AMPK activation might additionally benefit the dystrophic pathology by promoting muscle nitric oxide (NO) production. Using isolated mechanically stretched mouse cardiomyocytes, Garbincius and Michele, 2015 provided evidence that the DAPC functions as a mechanosensor to activate AMPK, which then phosphorylates nNOS to increase NO production in response to mechanical stress, and that loss of dystrophin disrupts this signaling mechanism. They also showed that acute pharmacologic activation of AMPK by AICAR or sodium salicylate was sufficient to restore neuronal nNOS activity and NO production in adult cardiomyocytes from *mdx* mouse.

Metformin is a widely used antidiabetic drug able to indirectly activate AMPK (Zhou et al., 2001). Treatment of *mdx* mice with metformin for 42 days has been shown to result in an increase of PGC1α and utrophin protein levels in TA muscle (Ljubicic and Jasmin, 2015). Successively, Mantuano and colleagues (Mantuano et al., 2018) tested the efficacy of metformin in *mdx* mice subjected to chronic treadmill exercise, a model of more severe metabolic condition that more closely mirrors human DMD pathology. Long-term metformin administration significantly reduced the structural damage and the levels of the fibrotic cytokine transforming growth factor β1 (TGFβ1) in gastrocnemius muscle and improved twitch and tetanic force in the diaphragm of *mdx* exercised mice. However, metformin did not significantly affect fiber type composition nor the transcript levels of utrophin and of genes involved in metabolic muscle adaptation to exercise and mitochondrial function and biogenesis. The lack of metabolic effect was attributed to the inability of metformin to increase the significantly reduced muscle content of NO precursors found in *mdx* muscle (Mantuano et al., 2018). Another study reported that the twitch and tetanic force of TA muscle from *mdx* mice treated with metformin for 90 days were significantly improved; this was accompanied by improvements in membrane integrity and neuromuscular transmission, and by an increase in the expression of DAPC components. Metformin was also observed to reduce muscle damage and fibrosis and enhance regeneration (Dong et al., 2021). Importantly, it has also been shown that AMPK activation by metformin decreases fibrosis in *mdx* mice by reprogramming macrophage phenotype. In fact, in fibrotic *mdx* muscles, pro-inflammatory macrophages exhibited a profibrotic activity by secreting high amounts of latent TGFβ1, which was subsequently activated by enzymes produced by fibroadipogenic progenitors (FAPs) that sustain production of collagen. AMPK activation skewed macrophage phenotype toward an anti-inflammatory profile, thus reducing TGFβ1 secretion by pro-inflammatory macrophages and fibrosis establishment, with a concomitant decrease in the necrotic area and an increase in the size of regenerating myofibers and muscle strength (Juban et al., 2018).

Metformin has been tested in clinical trials on DMD patients in combination with L-Arginine or L-Citrulline, an L-arginine precursor, to evaluate whether stimulation of AMPK and NO pathways are able to synergistically improve dystrophic muscle metabolism. In a pilot trial involving 5 DMD patients aged between 7 and 10 years (NCT02516085), a combined treatment of metformin and L-arginine resulted in decreased energy expenditure, energy substrate shift from carbohydrates to fatty acids, increased NO production and mitochondrial protein expression in muscle and decreased oxidative stress. These changes were associated with improved clinical scores (Hafner et al., 2016a). A subsequent single center, randomized, placebo-controlled trial evaluated the effect of a combined treatment with metformin and L-citrulline on the motor function decline in 47 ambulant children aged 6.5–10 years with DMD (NCT01995032). The treatment was well tolerated and resulted in a reduction of motor function decline that was however statistically significant only among a stable subgroup of patients (Hafner et al., 2016b; 2019).

To the aim of developing new, more translatable tools to activate AMPK, a recent study has examined the effects of MK-8722, a next-generation orally bioactive AMPK agonist, on the dystrophic pathology of *mdx* mice (Ng et al., 2023). A single dose of MK-8722 to *mdx* mice resulted in AMPK activation in TA muscle and initiated a disease-resistant gene expression program within 12 h post-treatment. Specifically, MK-8722 upregulated PGC1α activity and NRF1 mRNA and increased the protein levels and the extrasynaptic localization of utrophin. In parallel, MK-8722 increased AMPK-specific phosphorylation of Ulk1, an autophagy-initiating kinase, and downstream markers of autophagy signaling, as well as expression of transcription factor EB (TFEB)-targeted and autophagy-related genes. Furthermore, muscles from MK-8722-treated *mdx* mice displayed a reduced expression of the early differentiation marker myogenin and a greater number of quiescent satellite cells relative to untreated controls.

#### 4.1.3 SIRT1

AMPK controls the expression of genes involved in energy metabolism in skeletal muscle by acting in concert with the nicotinamide adenine dinucleotide (NAD+)-dependent type III deacetylase SIRT1. SIRT1 is activated in response to restriction of nutrients and influences numerous biological processes in skeletal and cardiac muscle by deacetylating various histones and several non-histone transcription regulators (Vinciguerra et al., 2010).

The activities of AMPK and SIRT1 are intimately linked. Indeed, AMPK indirectly activates SIRT1 by increasing intracellular NAD^+^ levels through the induction of mitochondrial fatty acid oxidation and by inducing the expression of nicotinamide phosphoribosyltransferase (Nampt), an enzyme of the NAD^+^ biosynthesis pathway (Fulco et al., 2008; Canto et al., 2009; Canto et al., 2010). This leads to deacetylation, and hence activation, of SIRT1 downstream targets that include PGC1α and FOXO transcription factors (Gerhart-Hines et al., 2007; Brunet et al., 2004), which are also targets for AMPK phosphorylation (Jäger et al., 2007; Greer et al., 2007). Notably, AMPK-mediated phosphorylation of PGC1α has been shown to serve as a priming signal for subsequent deacetylation by SIRT1, which provides evidence that these post-translational modifications of PGC1α by AMPK and SIRT1 are interconnected (Canto and Auwerx, 2009). Furthermore, the interplay between AMPK and SIRT1 might be reciprocal, as SIRT1 can activate AMPK through a global metabolic adaptation as well as via deacetylation and activation of the AMPK upstream kinase LKB1 (Feige et al., 2008; Lan et al., 2008).

Like AMPK, also SIRT1 has been recognized as a potent muscle phenotypic modifier and a potential target for DMD therapy (Kuno and Horio, 2016). In fact, muscle-specific transgenic overexpression of SIRT1 in healthy non-dystrophic mice has been shown to drive a shift to slow twitch oxidative muscle fiber type accompanied by increased levels of deacetylated PGC1α, an increase in mitochondrial content and activity, and increased expression of utrophin and NMJ genes. Similarly, SIRT1 transgenic overexpression in muscles of *mdx* mice resulted in enhanced expression of PGC1α, utrophin and NMJ genes, which was accompanied by attenuated muscle histopathology and improved physical performance (Chalkiadaki et al., 2014).

Resveratrol (RSV), a natural polyphenolic compound found in grapes and red wine, has been discovered in a small molecule screen to increase SIRT1 activity through an allosteric interaction (Howitz et al., 2003). Treatment of mice with RSV induces the aerobic capacity of the muscle, improves mitochondrial function and biogenesis, and induces a shift toward more oxidative muscle fibers (Lagouge et al., 2006). These actions of RSV appear to be primarily mediated through SIRT1, as assessed by using adult-inducible SIRT1 knockout mice (Price et al., 2012), although it has also been shown that many metabolic benefits of RSV *in vivo* require the induction of AMPK activity (Um et al., 2010). Regardless of which one is the primary target, RSV activates both SIRT1and AMPK, given their strict interdependence (Canto and Auwerx, 2010).

Several studies demonstrated that chronic RSV treatment can improve the dystrophic pathology in *mdx* mice, although the effectiveness of treatment was found to depend on the parameters of RSV administration such as the age of intervention, the RSV dose, and the duration of treatment. Hori et al. (2011) reported that muscles from *mdx* mice fed a diet supplemented with RSV (500 mg/kg/day) for 32 weeks starting at 9 weeks of age, displayed decreased oxidative damage and fibrosis, while infiltration of inflammatory cells and elevated levels of TGFβ1 were still observed. However, an *in vitro* analysis showed that pre-treatment of C2C12 myoblasts with resveratrol suppressed the TGFβ1-induced production of reactive oxygen species (ROS) and expression of fibroblastic markers, and SIRT1 knockdown abolished these effects (Hori et al., 2011). More precise administration of resveratrol through gavage allowed to determine that a dose of 100 mg/kg/day given to 5-week-old *mdx* mice for 10 days decreased immune cell infiltration in muscles at early stages of disease and increased SIRT1, PGC1α and utrophin mRNA expression (Gordon et al., 2013). However, a longer treatment period of 8 weeks improved rotarod performance and *in situ* peak tension and decreased central nucleation but had no effect on immune cell infiltration and oxidative capacity at 12 weeks of age (Gordon et al., 2014). Therefore, the anti-inflammatory effect of RSV was detected at early stages of disease, i.e., around the time of peak inflammation, but not at later stages when inflammation is already reduced. Consistently, when voluntary exercise was used to induce muscle damage and a greater inflammatory infiltrate, a long-term treatment (15 weeks) with a low dose of RSV(5 mg/kg/day) starting at 4 weeks of age was shown to be able to reduce exercise-induced necrosis and immune cell infiltration in the quadriceps muscle and gene expression levels of macrophage markers (Woodman et al., 2021). Furthermore, a report from Ljubicic et al. (2014b) highlighted the importance of the dosage regimen of resveratrol to maximize its potential effectiveness in counteracting *mdx* pathology. Fast glycolytic muscles from 6- to 7-week-old *mdx* mice fed a diet supplemented with a dose of RSV of 100 mg/Kg/day for 6 weeks displayed a significant increase in SIRT1 protein levels and activity, as well as a decrease in PGC1α acetylation, and an increased expression of PGC1α-target genes. These changes occurred in concomitance with a higher proportion of slower more oxidative fibers and were observed also in slow soleus muscle. However, a higher dose (500 mg/kg/day) and longer duration of RSV treatment (12 weeks) failed to stimulate SIRT1 and PGC1α activity and to promote expression of the slow oxidative myogenic program in *mdx* mouse muscle (Ljubicic et al., 2014b). In another study, 4 to 5-weeek-old *mdx* mice fed a diet supplemented with 100 mg/kg/day of RSV for 8 weeks displayed improved fatigue resistance in the soleus muscle, however, failed to increase resistance to contraction-induced injury in the soleus or EDL and did not achieve significant increases in utrophin expression (Selsby et al., 2012).

SIRT1 is known to promote autophagy by deacetylating and activating FOXO transcription factors, which induce the expression of autophagy-related genes (Brunet et al., 2004). SIRT1 also deacetylates directly several autophagy-related proteins (Lee et al., 2008). It has been reported that autophagy/mitophagy-related genes and autophagic flux were downregulated in muscles of *mdx* mice and that exposure to a diet supplemented with various doses of RSV for 56 weeks, starting at 9 weeks of age, was able to rescue such defects and to reduce ROS production. Furthermore, RSV treatment decreased myofiber wasting and serum CK levels, and significantly increased the animal’s physical activity (Sebori et al., 2018).

Other studies from the Horio’s group have reported beneficial effects of RSV on the cardiac pathophysiology of *mdx* mice (Kuno et al., 2013; Kuno et al., 2018). Indeed, treatment with an RSV-supplemented diet (500 mg/kg/day) for 32 weeks starting at 9 weeks of age inhibited cardiac hypertrophy and fibrosis and improved cardiac function in the *mdx* heart and concomitantly downregulated the protein levels of p300, an acetyltransferase that promotes adaptive cardiac hypertrophy (Kuno et al., 2013). A longer-term exposure (56 weeks) to a diet containing increasing doses of RSV promoted mitophagy and reduced oxidative stress in the *mdx* mouse heart by facilitating the degradation of autophagosomes containing damaged mitochondria. Furthermore, RSV induced SIRT1 and FOXO3a activity and the expression of autophagy-related downstream genes and improved pathophysiological conditions of the hearth. The maximum benefit was obtained by an RSV dosage of 50 mg/kg/day (Kuno et al., 2018). The effects of resveratrol supplementation were also tested in an open-label, single-arm, pilot phase IIa trial in 11 patients with Duchenne, Becker or Fukuyama muscular dystrophies (Kawamura et al., 2020). Although this study was quite small, it showed significant improvements in motor function and reduction of serum CK levels.

In addition to RSV, quercetin, a flavonoid with anti-inflammatory and antioxidant properties, has been identified as a direct activator of SIRT1 (Howitz et al., 2003). Hollinger and colleagues (Hollinger et al., 2015) found that *mdx* mice fed a quercetin-enriched diet for 6 months beginning at 3 months of age displayed decreased histopathological injury in diaphragm muscle, including decreased fibrosis and immune cell infiltration and decreased number of centrally nucleated fibers. The quercetin-enriched diet increased mRNA expression of genes associated with oxidative metabolism but did not increase utrophin mRNA or protein expression (Hollinger et al., 2015). Strikingly, however, another study showed that dietary quercetin enrichment improved the respiratory function in *mdx* mice only for the first 4–6 months during a 12-month dosing regimen, but no improvement of diaphragm histopathology or increase of downstream effectors of the SIRT1/PGC1α pathway were observed at the end of the 12-month treatment period, suggesting the development of an age-dependent quercetin insensitivity (Selsby et al., 2016). Similarly, histopathology was not significantly improved in hindlimb muscles of *mdx* mice fed a quercetin-supplemented diet for 12 months, except for an increase in specific tension and partially preserved fatigue resistance observed in the soleus muscle (Spaulding et al., 2016). The effects of quercetin were also tested in the D2-*mdx* mouse model of DMD that has a more severe dystrophic phenotype than *mdx* mice (Coley et al., 2016). It was found that a 7-month treatment with quercetin alone or in combination with nicotinamide riboside (a NAD^+^ donor) and lisinopril (a cardioprotective drug) did not prevent diaphragmatic and hindlimb injury or preserve respiratory function in the D2-*mdx* mice (Spaulding et al., 2019; Spaulding et al., 2020b). Therefore, the efficacy of quercetin in countering dystrophic skeletal muscle pathology appears limited. However, Ballmann and colleagues provided evidence that dietary quercetin enrichment may counter cardiac complications associated with DMD (Ballmann et al., 2015; Ballmann et al., 2017a; Ballmann et al., 2017b). Specifically, a 6-month quercetin treatment increased mitochondrial biogenesis, antioxidant enzyme abundance and utrophin expression in association with attenuated fibrotic damage in hearts from young *mdx* mice (Ballmann et al., 2015). Another study highlighted the cardio-protective effect of quercetin in *mdx*/Utrn^+/−^ mice, a less studied model of murine DMD that exhibits an exacerbated pathology. In fact, *mdx*/Utrn^+/−^ mice fed a quercetin-enriched diet for 8 months displayed improved cardiac function as well as decreased inflammation and fibrosis and increased expression of utrophin and various mitochondrial markers and antioxidant enzymes in hearth tissues (Ballmann et al., 2017a). Similar improvements in cardiac histological and biochemical parameters, but not function, were observed in *mdx* mice fed a quercetin-enriched diet for 8 months (Ballmann et al., 2017b).

#### 4.1.4 PPARβ/δ

Peroxisome proliferator-activated receptors (PPARs), which are members of the nuclear receptor superfamily of transcription factors, act as fatty acid sensors to control many metabolic programs that are essential for energy homeostasis (Wang, 2010). Among the three isotypes of PPARs (α, β/δ and γ) PPARβ/δ predominates in skeletal muscle, where it shows a higher expression in slow-oxidative muscle fibers compared to glycolytic type II muscle fibers (Wang et al., 2004). The use of synthetic agonists and muscle-specific gain and loss of function mouse models has revealed the crucial role played by PPARβ/δ in muscle physiology and energy metabolism (Ehrenborg and Krook, 2009). Specifically, activation of PPARβ/δ increases mitochondrial activity and enhances resistance to fatigue and running endurance by promoting fast to slow oxidative myofiber type switch and a metabolic shift in energy substrate from glucose to fatty acid (Wang et al., 2004; Luquet et al., 2003; Chen et al., 2015; Fan et al., 2017). Furthermore, PPARβ/δ stimulates the expression of PGC1α, which in turn synergistically coactivates PPARβ/δ in a ligand-dependent manner (Schuler et al., 2006; Kleiner et al., 2009; Koh et al., 2017).

It has been shown that PPARβ/δ protein is expressed at higher levels in both slow and fast muscles of *mdx* mice compared to healthy mice and that its pharmacological activation using the selective agonist GW501516 ameliorates the dystrophic pathology. Indeed, a 4-week GW501516 oral treatment given to *mdx* mice beginning at 5–7 weeks of age increased the number of slower more oxidative myofibers, enhanced expression of utrophin A and members of the DAPC in extrasynaptic regions of the muscle membrane, and improved sarcolemmal integrity. GW501516 treatment also conferred protection against contraction-induced damage to *mdx* skeletal muscle. Furthermore, *in vitro* studies using C2C12 myoblasts revealed that PPARβ/δ activation by GW501516 can stimulate utrophin A mRNA levels through direct activation of the utrophin A promoter (Miura et al., 2009). In another study, 12-week-old *mdx* mice were treated with the PPARβ/δ agonist GW501516, alone or in combination with the AMPK agonist AICAR, for 4 weeks. A significant increase in utrophin A protein levels, mitochondrial content, oxidative myofibers and forelimb and hindlimb grip strength was found in the GW501516-treated and combination treatment groups along with significant improvements in disease phenotype, including a decrease in muscle inflammation and fibrosis as well as in the number of fibers with central nuclei and regenerating fibers (Jahnke et al., 2012).

GW501516 entered clinical trials as a drug candidate for metabolic and cardiovascular disorders, but further development was abandoned due tumorigenic effects observed in long-term (2 years) carcinogenesis studies in mice and rats (Geiger et al., 2009; Newsholme et al., 2009). Recently, a series of novel PPARβ/δ modulators with increased specificity and improved side-effect profile compared to GW501516 have been discovered (Wu et al., 2017; Lagu et al., 2018a). The compound MA-0204 has been shown to specifically increase PPARβ/δ target genes and improve the utilization of fatty acids in myoblasts isolated from a DMD patient (Lagu et al., 2018b). Furthermore, treatment of myoblasts isolated from *mdx* mice with a similar compound (MTB-6) has been shown to specifically induce expression of PPARβ/δ target genes and to increase ATP flux and expression levels of genes specifically involved in fatty acid oxidation (Bell et al., 2019). Currently the PPARβ/δ specific agonist ASP0367 (also known as Docidelpar, and formerly as MA-0211 or MTB-1) is being advanced into human trials. The results of a phase 1 trial (NCT03682484) have shown ASP0367 upregulates PPARβ/δ target genes, has a good safety profile and is well tolerated in healthy adults (Ito et al., 2022). Furthermore, a clinical study aiming to learn how ASP0367 is processed by the body of adult heathy men is underway (NCT05217901). ASP0367 is also being assessed in a phase 1b study in pediatric male patients with DMD (NCT04184882), but this study was terminated early due to enrollment challenges and the small sample size limits the data interpretation.

#### 4.1.5 ERRγ

The estrogen receptor-related receptor γ (ERRγ), belonging to the estrogen-related receptor subfamily of nuclear receptors, is mainly expressed in metabolically active and highly vascularized tissues. In skeletal muscle tissue, ERRγ is exclusively and abundantly expressed in oxidative and slow-twitch muscles and, when transgenically expressed in fast-twitch glycolytic muscles, simultaneously activates metabolic and vascular pathways specific to oxidative fibers. In fact, muscle-specific ERRγ transgenic mice exhibited a shift toward oxidative/slow fiber type and enhanced mitochondrial function, myofibrillar neo-vascularization, and running endurance (Rangwala et al., 2010; Narkar et al., 2011). These effects of ERRγ correlated with activation of AMPK, but not PGC1α (Narkar et al., 2011). ERRγ has also been shown to promote muscle repair via metabolic/fiber type and angiogenic remodeling in an ischemia model of muscle injury (Matsakas et al., 2012).

The expression of ERRγ was found to be downregulated in muscles of *mdx* mice compared with WT mice, and muscle-specific transgenic induction of ERRγ restored the expression of both oxidative and angiogenic genes that were repressed in dystrophic muscle (Matsakas et al., 2013). Furthermore, transgenic overexpression of ERRγ in *mdx* muscle resulted in enhanced mitochondrial biogenesis, an increased proportion of oxidative myofibers, and improved exercise tolerance. This correlated with enhanced muscle vasculature and improved blood flow. The improvement of metabolic and angiogenic features mediated by ERRγ in *mdx* mice mitigated the dystrophic pathology, as indicated by reduced myofiber damage and reduced numbers of regenerated myofibers. Similarly, ERRγ overexpression was shown to protect dystrophic muscle against post-exercise damage and hypoxia. Remarkably, ERRγ upregulation did not result in increased sarcolemmal utrophin expression or sarcolemmal restoration of DAPC components, indicating that the beneficial effects elicited by ERRγ are mainly due to improved oxidative capacity and enhanced muscle angiogenesis (Matsakas et al., 2013).

### 4.2 Repressors of the slow/oxidative myogenic program

#### 4.2.1 Fnip1

Folliculin interacting protein-1 (Fnip1) is an intracellular protein that interacts with folliculin, Fnip2 and all three subunits (α, β, γ) of AMPK (Baba et al., 2006).

It has been shown that Fnip1 is predominantly expressed in skeletal muscles rich in glycolytic type IIb fibers where it functions as a negative regulator of the slow oxidative myogenic program. Indeed, Fnip1-null mice exhibited a shift to slow and highly oxidative fibers (type I and type IIa), increased numbers of highly functional mitochondria, increased resistance to fatigue and more rapid post-contraction recovery. Furthermore, muscles from Fnip1-null mice displayed increased activation of AMPK and PGC1α and genetic ablation of PGC1α greatly reduced the expression of slow-oxidative fiber markers, indicating that PGC1α is an essential mediator of the oxidative fiber type shift seen in Fnip1-null muscle. Remarkably, genetic disruption of Fnip1 in *mdx* mice significantly reduced muscle fiber damage and compensatory regeneration without affecting the expression of utrophin (Reyes et al., 2015).

Interestingly, the microRNA miR-499, embedded in an intronic region of the *Myh7b* gene encoding slow type I MyHC, has been identified as a negative regulator of Fnip1, thus revealing a miR-499/Fnip1/AMPK circuit that couples muscle fiber type and mitochondrial oxidative metabolism (Liu et al., 2016). Indeed, skeletal muscle-specific transgenic overexpression of miR-499 resulted in downregulation of Fnip1 protein levels and a dramatic increase of slow type I muscle fibers and mitochondrial oxidative metabolism capacity through the activation of AMPK-PGC1α signaling, a phenotype that substantially recapitulates that of Fnip1-null mice. The levels of miR-499 were found to be downregulated in muscles of *mdx* mice and the transgenic restoration of miR-499 expression resulted in downregulation of Fnip1, increased expression of PGC1α, and activation of the slow-oxidative muscle fiber program. This correlated with a significant improvement of the pathophysiology of the muscular dystrophic phenotype, as indicated by a decrease of damaged and regenerated myofibers, reduced serum CK levels, and enhanced treadmill exercise performance (Liu et al., 2016). While the above studies suggests that Fnip1 functions as a negative regulator of AMPK, a recent study has demonstrated that AMPK negatively regulates Fnip1 through direct phosphorylation suppressing the function of Fnip1, and that Fnip1 phosphorylation is required for AMPK to induce nuclear translocation of TFEB and TFEB-dependent increase of PGC1α (Malik et al., 2023). These findings suggest the existence of a negative feedback loop between Fnip1 and AMPK.

#### 4.2.2 E2F1

Several evidence has revealed that cell cycle regulatory proteins can also modulate metabolic pathways both in physiological conditions that require changes in cellular metabolism and in pathological conditions (Fajas, 2013).

In addition to its well-described role in cell cycle progression, the E2F1 transcription factor participates in the differentiation of several tissues and regulates specific metabolic functions in fully differentiated organs involved in global energy homeostasis (Denechaud et al., 2017). In skeletal muscle and brown adipose tissue (BAT), E2F1 has been shown to negatively regulate oxidative metabolism in basal conditions by forming a repressor complex with the retinoblastoma protein (pRb) on the promoters of genes involved in oxidative metabolism and mitochondrial function. Consequently, E2F1^−/−^ mice displayed increased energy expenditure and thermogenesis, increased mitochondrial DNA content and function and enhanced expression of oxidative genes in BAT and muscle. These changes correlated with an increased proportion of slow-oxidative muscle fibers and better resistance to fatigue during exercise (Blanchet et al., 2011). Importantly, E2F1 was found upregulated in muscles of *mdx* mice and DMD patients. Genetic inactivation of E2F1 in *mdx* mice resulted in a significant attenuation of physiopathological signs of dystrophic disease, as indicated by decreased muscle damage, regeneration and inflammation, increased numbers of slower oxidative fibers and utrophin protein expression, increased oxidative metabolic gene program, and improved muscle function (Blanchet et al., 2012).

#### 4.2.3 Cyclin D3

The canonical function of D-type cyclins is to promote progression through the G1 phase of the cell cycle by activating the partner cyclin-dependent kinases 4 and 6 (CDK4/6) that phosphorylate and inhibit pRb. Besides pRb, cyclinD-CDK4/6 complexes target other proteins for phosphorylation, with considerable difference in substrate specificity depending on the associated D type cyclin (Anders et al., 2011), and D cyclins can also perform kinase-independent transcriptional functions (Hydbring et al., 2016). Remarkably, it was reported that CDK4/CyclinD3 complexes specifically phosphorylate and inhibit the catalytic α2 subunit of AMPK and that mice treated with a CDK4 inhibitor have increased oxidative metabolism (Lopez-Mejia et al., 2017).

Studies from our laboratory have revealed specific roles played by cyclin D3 in the control of skeletal muscle precursor cell proliferation and terminal differentiation both *in vitro* and *in vivo* (Cenciarelli et al., 1999; De Santa et al., 2007; De Luca et al., 2013). Furthermore, we uncovered a novel cell cycle-independent function of cyclin D3 in regulating muscle fiber type phenotype and whole-body energy metabolism. Indeed, cyclin D3 knockout mice displayed a shift in skeletal muscle fibers toward a slower, more oxidative phenotype, enhanced exercise capacity and increased energy expenditure and fatty acid oxidation. In addition, a transcriptomic analysis of cyclinD3-null muscle revealed the upregulation of genes characteristic of the slow-oxidative myogenic program, many of which are targets of MEF2 and or NFAT transcription factors (Giannattasio et al., 2018). These observations prompted us to test whether cyclin D3 inactivation protects from muscular dystrophy. Genetic inactivation of cyclin D3 in *mdx* mice resulted in a higher proportion of slower, more oxidative muscle fibers with a concomitant reduction of degenerative/regenerative processes, myofiber size variability, and muscle fatigability, indicating an attenuation of dystrophic pathology. Furthermore, *mdx*/cyclin D3-null mice exhibited better performance in repeated trials of treadmill exercise compared with *mdx* mice and their muscles showed decreased exercise-induced damage, enhanced regenerative capacity and increased mRNA expression of genes involved in the control of oxidative metabolism and the response to oxidative stress (Bonato et al., 2023).

#### 4.2.4 Nfix

The transcription factor Nfix can act as a transcriptional activator and/or repressor. During pre-natal development, NFix is expressed in fetal muscle where it regulates the switch from embryonic to fetal myogenesis by indirectly repressing the expression of embryonic (slow) genes while activating the fetal (fast) myogenic gene program (Messina et al., 2010; Taglietti et al., 2016). In adult skeletal muscle, Nfix has been shown to regulate the temporal progression of the regeneration process through modulation of myostatin expression. Specifically, adult muscles from Nfix-null mice were characterized by a reduced muscle fiber size, increased expression of the slow MyHC isoform, and delayed regeneration in response to acute damage (Rossi et al., 2016). This led to the hypothesis that ablation of Nfix may exert a beneficial effect on the dystrophic pathology. Indeed, ablation of Nfix in the dystrophic *mdx* and α-sarcoglycan (*sgca*)-deficient mouse models resulted in a shift to a slow, more oxidative musculature, delayed regeneration and improved muscle histopathology and muscle performance. It was suggested that delaying regeneration benefits dystrophic muscle because of a slowdown of the repetitive cycles of muscle degeneration and regeneration (Rossi et al., 2017). In addition, specific deletion of Nfix in macrophages of sgca-null dystrophic mice has been shown to protect from muscle wasting, delaying the establishment of FAP-dependent fibrosis (Saclier et al., 2022).

The concept that delaying regeneration may attenuate dystrophic pathology is corroborated by a recent study which has formally demonstrated that persistent activation of satellite cells directly contributes to muscular dystrophy pathogenesis and progression (Boyer et al., 2022). In fact, it was found that genetical ablation of satellite cells in neonatal or young adult dystrophic mice (*mdx* and delta-sarcoglycan-deficient mice) improved histopathology and muscle performance and reduced the muscle susceptibility to contraction-induced injury. Mechanistically, it was shown that the induction of MyoD in activated satellite cells leads to re-expression of the fetal gene program in dystrophic muscle fibers, which destabilizes the myofiber sarcolemma with consequent vulnerability to damage (Boyer et al., 2022).

#### 4.2.5 RAGE

RAGE (receptor for advanced glycation end-products) is a multiligand receptor of the immunoglobulin superfamily that mediates several physiological and pathological processes, including tissue regeneration/repair, inflammation, and cancer, through the activation of multiple intracellular signaling cascades (Riuzzi et al., 2018). In skeletal muscle, RAGE is expressed during development and repressed in the adulthood. However, RAGE is transiently re-expressed in activated satellite cells and regenerating myofibers upon acute muscle injury and critically regulates the appropriate timing of muscle regeneration and the homeostasis of muscle precursor cells (Riuzzi et al., 2018). Furthermore, RAGE is re-expressed and over-stimulated in atrophying myofibers of tumor-bearing mice,and genetic ablation of RAGE provides resistance to tumor-induced muscle atrophy (Chiappalupi et al., 2020).

RAGE may play a role in myofiber type transition, as evidenced by the high prevalence of slow type I MyHC and the remarkably low levels of fast MyHC isoforms in muscles of RAGE-null mice (Chiappalupi et al., 2020). RAGE and RAGE ligand expression was shown to be elevated in muscles of *mdx* mice, suggesting that chronic activation of RAGE sustains inflammation and contributes to muscle degeneration during disease progression. Indeed, genetic inactivation of RAGE in *mdx* mice or treatment of dystrophic muscles with a blocking anti-RAGE antibody resulted in restrained inflammation, lower percentages of necrotic myofibers, reduced recruitment of muscle precursor cells, and improved muscle strength (Sagheddu et al., 2018).

## 5 Conclusion

The therapeutic approaches for DMD that are under development can be divided into two categories: those aiming at correcting the primary genetic defect and those targeting downstream determinants of disease progression. Dystrophin restoration therapies hold great promise but still have low targeting efficiency in fibrotic dystrophic muscle. Thus, treatments that protect muscle from progressive and irreversible deterioration are crucial not only to preserve muscle quality and function for as long as possible but also to improve the therapeutic efficacy of gene-based and cell-based treatment approaches.

Preclinical studies highlighted in this review and summarized in Table 1 clearly show that the pathology of DMD can be alleviated by activating factors like PGC1α, PPARδ, ERRγ, AMPK, and SIRT1 that promote remodeling of skeletal muscle towards the slow/oxidative phenotype, which is more resistant to contraction-induced damage, or by inactivating factors like Fnip1, E2f1, cyclin D3, Nfix, and RAGE that repress this phenotype. This attenuation may be partially attributed to the upregulation of utrophin, which is typically expressed at higher levels in slow oxidative fibers compared with fast glycolytic fibers. Furthermore, many of these phenotypic remodeling factors have been shown to beneficially alter DMD pathology by stimulating autophagy/mitophagy, mitochondrial biogenesis and function, and by inhibiting fibrosis and oxidative stress. Therefore, their pharmacological modulation may be a useful therapeutic strategy to counteract multiple secondary pathological mechanisms caused by dystrophin deficiency. It may also be used in conjunction with other therapeutic strategies that target the primary genetic defect for a synergistic therapeutic effect.

**TABLE 1:** Modulation of activators or repressors of the slow/oxidative myogenic program in preclinical studies for DMD treatment.

Therapeutic strategy	Experimental model	Effects	References
AMPK activation	5 to 7-week-old *mdx* mice treated with AICAR (500 mg/Kg/day) by subcutaneous injection for 30 days	↑MyHCIIa-positive fibers and COX activity in EDL muscle ↓contraction-induced damage in EDL ↑utrophin protein and β-DG expression along the sarcolemma in EDL ↑PGC1α protein levels	Ljubicic et al. (2011)
AMPK activation	12-week-old *mdx* mice treated with AICAR (250 mg/Kg/day) by IP injection, 5 days/week for 4 weeks	↑mitochondrial activity in *mdx* primary myoblasts ↑EDL and soleus muscle oxidative capacity ↑MyHC1 fibers in soleus ↑EDL twitch force (TTP) ↓regenerating fibers, damaged fibers and centronucleated fibers in GAS ↑levels of utrophin protein in EDL ↓fibrosis in diaphragm	Jahnke et al. (2012)
AMPK activation	3 to 4-week-old *mdx* mice treated with AICAR (500 mg/Kg/day) by subcutaneous injection for 28 days	↑MyHCIIa-positive fibers and oxidative capacity in EDL ↓muscle damage and central nucleation in EDL ↑expression of utrophin and β-DG ↑forelimb muscle strength	Al-Rewashdy et al. (2015)
AMPK activation	3 to 4-week-old utrophin-deficient *mdx* mice treated with AICAR (500 mg/Kg/day) by subcutaneous injection for 28 days	↑MyHCIIa-positive fibers and oxidative capacity in EDL	Al-Rewashdy et al. (2015)
AMPK activation	6-week-old *mdx* mice treated with AICAR (500 mg/Kg/day) by IP injection for 28 days	↑autophagic pathway (Ulk1, Beclin-1, LC3II/LC3I ratio, Bnip3) in diaphragm ↑mitochondrial PTP function ↑MHC1(slow) fibers and mitochondrial complex I in the diaphragm ↓centronucleated fibers ↑diaphragmatic force production	Pauly et al. (2012)
AMPK activation	Adult *mdx* cardiomyocytes stretched *in vitro* treated with AICAR (2 mM) or Sodium Salycilate (10 mM) for 60 min	↑NO production ↑activity of AMPK and nNOS	Garbincius and Michele (2015)
AMPK activation	6-week-old *mdx* mice treated with Metformin (2 mg/ml in drinking water) for 42 days (average dose: 285.6 mg/Kg/day)	↑PGC1α and utrophin protein levels in TA muscle	Ljubicic and Jasmin (2015)
AMPK activation	4 to 5-week-old **exercised** *mdx* mice treated with Metformin (200 mg/Kg/day in drinking water) for 20 weeks	↑diaphragm twitch and tetanic force ↓muscle damage in GAS ↓levels of TGFβ1 (pro-fibrotic marker) in GAS	Mantuano et al. (2018)
AMPK activation	5 to 6-week-old *mdx* mice treated with Metformin (200 mg/Kg/day by IP injection) for 90 days	↑forelimb grip strength ↑twitch and tetanic force in TA muscle ↓percentage of damaged and fibrotic area in TA muscle ↑centrally-nucleated and newly generated fibers in TA ↑membrane integrity in TA ↑post-synaptic neuromuscular transmission in EDL ↑expression of DAPC components	Dong et al. (2021)
AMPK activation	8-10-week-old Fib-*mdx* mouse model, with injury induced over 2 weeks in TA muscle, treated with Metformin (200 mg/Kg/day in drinking water) for 3.5 weeks	↓production of latent TGFβ1 by macrophages ↓necrotic and fibrotic areas in TA ↑CSA of regenerating fibers ↓percentage of inflammatory macrophages ↑percentage of anti-inflammatory macrophages ↑isometric force production in TA muscle	Juban et al. (2018)
AMPK activation	10-week-old *mdx* mice treated with MK-8722 (1 mg/mL) via gavage for 3, 6, 12 h	↑PGC1α and NRF1 mRNA ↑nuclear accumulation of PGC1α protein in EDL ↑expression of extra-synaptic utrophin ↑phosphorylation of Ulk1 in TA ↑LC3II/LC3I ratio and p62 protein levels in TA ↑nuclear accumulation of TFEB protein in EDL ↑mRNA of TFEB targeted and autophagy-related genes in TA ↓activation of satellite cells	Ng et al. (2023)
Cyclin D3 inactivation	5-, 8-, and 20-week-old Cyclin D3-null/*mdx* mice	↑slower, oxidative fibers in TA and EDL muscles ↓muscle damage, regeneration, and myofiber size variability in TA muscle ↓fatigability of TA muscle ↑exercise tolerance and post-exercise regeneration ↓post-exercise damage in GAS muscle	Bonato et al. (2023)
E2F1 inactivation	8-week-old E2F1-null/*mdx* mice	↑slower oxidative fibers in various muscles ↑expression of utrophin and DAPC members in GAS muscle ↑oxidative gene expression in GAS ↓damage, regeneration, inflammation in various muscles ↓fiber size variability in GAS muscle ↓serum CK levels ↑force production in diaphragm at 12 weeks of age ↑treadmill exercise performance	Blanchet et al. (2012)
ERRγ upregulation	6 to 8-week-old α-skeletal actin-ERRγ/*mdx* mice	↑expression of oxidative and angiogenic genes in quadriceps muscle ↑slower, oxidative myofibers in TA ↑mitochondrial biogenesis in GAS and quadriceps ↑angiogenesis in TA ↓levels of serum CK ↓myofiber damage and regeneration in TA and GAS ↑exercise capacity ↓post-exercise damage and hypoxia in GAS and TA	Matsakas et al. (2013)
Fnip1 inactivation	8 to 12-week-old Fnip1-null/*mdx* mice	↓muscle damage in GAS ↓centronucleated fibers in quadriceps muscle ↓serum CK levels	Reyes et al. (2015)
Fnip1 inactivation	5 to 9-week-old MCK-miR-499/*mdx* mice	↑levels of PGC1α and myoglobin protein in GAS ↓muscle damage and centronucleated fibers in TA ↑MyHC1 fibers in the soleus ↓serum CK levels ↑exercise performance	Liu et al. (2016)
Nfix inactivation	3-, 5-, 8-, and 12-week-old Nfix-null/Sgca-null mice	↓damage, inflammation and fibrosis in TA and diaphragm ↑oxidative fibers in TA muscle ↓centronucleated fibers in TA and diaphragm ↓regeneration ↑exercise performance	Rossi et al. (2017)
Nfix inactivation	10-week-old Nfix-null/*mdx* mice	↓damage in TA and GAS muscles ↓inflammation ↓centronucleated fibers in TA and GAS muscles	Rossi et al. (2017)
PGC1α upregulation	4 to 6-week-old *mdx*/MCK- PGC1α mice	↓myofiber damage in sedentary and exercised conditions ↓centrally-nucleated myofibers ↓serum CK levels ↑ exercise tolerance ↑utrophin mRNA and protein	Handschin et al. (2007a)
PGC1α upregulation	5-week-old *mdx*/MCK- PGC1α mice	↓myofiber damage in sedentary and exercised conditions ↓centrally-nucleated myofibers and myofiber size variability ↑exercise tolerance ↑levels of utrophin mRNA and protein ↑mRNA levels of NMJ and Ox-Phos genes	Chan et al. (2014)
PGC1α upregulation	10-week-old *mdx/utrn* ^*−/−*^ */*MCK-PGC-1α mice	↓serum CK levels ↓myofiber damage ↑mRNA levels of NMJ and Ox-Phos genes	Chan et al. (2014)
PGC1α upregulation	*mdx*/MCK-Ind_PGC1α *(mice were switched to chow lacking doxycyline at 3 weeks of age and examined 4 weeks later)*	↑mRNA levels of utrophin, NMJ and Ox-Phos genes ↓ serum CK levels ↓myofiber damage ↓centrally-nucleated myofibers	Chan et al. (2014)
PGC1α upregulation	AAV-mediated PGC1α gene transfer in limbs of neonatal *mdx* mice *(mice were examined at 4–6 weeks and 6 months of age)*	↑resistance to contraction-induced muscle damage and fatigue ↑utrophin protein ↑type I slow fibers in the soleus ↑expression of oxidative and mitochondrial proteins (myoglobin, cytochrome C, UCP-1, complex IV subunits)	Selsby et al. (2012)
PGC1α upregulation	Plasmid-mediated PGC1α gene transfer in muscles of 6-week-old *mdx* mice *(mice were examined 7* *days later)*	↑activity of mitochondrial marker enzymes ↓resistance to PTP opening in response to Ca^2+^ overload ↑activity of calpain, caspase 3 and caspase 9	Godin et al. (2012)
PGC1α upregulation	AAV-mediated PGC1α gene transfer in muscles of 3-week-old *mdx* mice *(mice were examined at 6-week of age)*	↓muscle damage ↑utrophin protein expression ↑resistance to contraction-induced fatigue and injury in soleus ↑mRNA levels of DAPC components, and genes involved in oxidative metabolism, autophagy and satellite cell activation ↑TFEB nuclear localization ↑markers of lysosome biogenesis and autophagosome formation	Hollinger et al. (2015) Spaulding et al. (2020a)
PGC1α upregulation	AAV-mediated PGC1α gene transfer in muscles of 12-month-old *mdx* mice *(mice were examined at 15* *months of age)*	↑resistance to fatigue and specific tension of soleus	Hollinger and Selsby, 2015
PPARβ/δ activation	5–7-week-old *mdx* mice treated with GW501516 (25 mg/Kg/day) via gavage for 4 weeks	↑MyHC type1 fibers in soleus ↑MyHC type2a fibers in EDL ↑uthrophin A and DAPC members at the sarcolemma in soleus ↑utrophin mRNA in soleus and EDL ↓myofiber membrane damage in soleus ↓contraction-induced force drop in EDL	Miura et al. (2009)
PPARβ/δ activation	12-week-old *mdx* mice treated with GW501516 (7.5 mg/Kg/day) via gavage, 5 days/week, for 4 weeks	↑ expression of uthrophin A proteinin EDL ↑mitochondrial content in mdx primary myoblasts ↑EDL and soleus muscle oxidative capacity ↑number of MyHCIIa myofibers in soleus ↑grip strength ↑twitch force parameters in EDL ↓myofiber damage and regeneration in GAS ↓fibrosis in diaphragm ↓inflammatory infiltrate in EDL	Jahnke et al. (2012)
PPARβ/δ activation	Primary myoblasts isolated from muscle of a 17-month-old DMD patient treated with increasing concentrations of MA-0204 for 48 h	↑expression of PPARβ/δ target genes ↑fatty acid oxidation	Lagu et al. (2018b)
PPARβ/δ activation	Primary myoblasts isolated from muscles of *mdx* mice treated with MTB-6 (800 nM) for 48h	↑ATP/AMP ratio ↑levels of genes involved in fatty acid oxidation	Bell et al. (2019)
RAGE inactivation	5-week-old RAGE-null/*mdx* mice	↓necrotic myofibers ↓inflammation ↓CSA of regenerating myofibers ↓recruitment of muscle progenitors ↑muscle strenght	Sagheddu et al. (2018)
SIRT1 upregulation	8- to 10-week-old MCK-SIRT1/*mdx* mice	↑mRNA levels of PGC1α, utrophin and NMJ genes in GAS ↓damaged area in GAS ↓serum CK levels ↑running performance	Chalkiadaki et al. (2014)
SIRT1 activation	9-week-old *mdx* mice fed RSV-supplemented diet (4 g/Kg food) for 32 weeks *(estimated RSV dose:500 mg/Kg/day)*	↓loss of muscle mass ↓oxidative damage ↓fibrosis	Hori et al. (2011)
SIRT1 activation	5-week-old *mdx* mice treated with RSV (10-100-500 mg/Kg/day) via oral gavage for 10 days	↑expression of SIRT1, PGC1α and utrophin mRNA in GAS ↓inflammatory cell infiltration in GAS	Gordon et al. (2013)
SIRT1 activation	4- to 5-week-old *mdx* mice treated with RSV (100 mg/Kg) via oral gavage every other day for 8 weeks	↑rotarod performance and *in situ* peak tension ↓central nucleation	Gordon et al. (2014)
SIRT1 activation	4-week-old *mdx* mice fed RSV-supplemented diet (5 mg/Kg/day) for 15 weeks	↓exercise-induced damage in GAS muscle ↓mRNA expression of macrophage markers	Woodman et al. (2021)
SIRT1 activation	6–7-week-old male *mdx* mice fed RSV-supplemented diet (100 mg/Kg/day) for 6 weeks	↑SIRT1 levels and activity in EDL and soleus ↓acetylation of PGC1α in GAS ↑expression of PGC1α target genes (COX IV) in EDL and soleus ↑numbers of slower oxidative fibers in both EDL and soleus muscle	Ljubicic et al. (2014b)
SIRT1 activation	1 month-old *mdx* mice fed RSV-supplemented diet (100 mg/Kg/day) for 8 weeks	↑fatigue resistance in soleus muscle ↓muscle weights	Selsby et al. (2012)
SIRT1 activation	9-week-old *mdx* mice fed RSV-supplemented diet (0.04, 0.4, and 4 g/kg food) for 56 weeks *(estimated RSV dose: 5, 50 and 500 mg/Kg/day)*	↑mRNA of autophagy and mitophagy- related genes in quadriceps ↓ levels of ubiquitinated proteins in quadriceps ↓ROS levels in quadriceps ↓central nucleation and increased CSA in quadriceps ↓serum CK levels ↑physical activity	Sebori et al. (2018)
SIRT1 activation	9-week-old *mdx* mice fed RSV-supplemented diet (4 g/Kg food) for 32 weeks *(estimated RSV dose: 500 mg/Kg/day*	↓cardiac hypertrophy and fibrosis ↓histone H3 acetylation ↓of p300 acetyltransferase protein levels ↑cardiac function	Kuno et al. (2013)
SIRT1 activation	9-week-old *mdx* mice fed RSV-supplemented diet (0.04, 0.4, and 4 g/kg food) for 56 weeks *(estimated RSV dose: 5, 50 and 500 mg/Kg/day)*	↑mitophagy in the hearth ↓oxidative stress in the hearth ↑activity of SIRT1 and FOXO3a ↑expression of autophagy-related downstream genes ↓fibrosis	Kuno et al. (2018)
SIRT1 activation	12-week-old *mdx* mice fed 0.2% quercetin-enriched diet for 6 months	↓central nucleation, immune cell infiltration and fibrosis in diaphragm ↑mRNA expression of oxidative genes in diaphragm	Hollinger et al. (2015)
SIRT1 activation	8-week-old *mdx* mice fed 0.2% quercetin-enriched diet for 12 months	↑respiratory function during the first 4–6 months of treatment ↑specific tension in soleus ↑spontaneous activity	Selsby et al. (2016) Spaulding et al. (2016)
SIRT1 activation	3-week-old or 12-week-old *mdx* mice fed 0.2% quercetin-enriched diet for 6 months	↑mitochondrial density, SOD2 protein abundance and utrophin protein expression in hearth ↓MMP9 protein expression ↓cardiac fibrosis	Ballmann et al. (2015)
SIRT1 activation	8-week-old *mdx*/Utrn ± mice fed 0.2% quercetin-enriched diet for 8 months	↓damage, inflammatory markers and fibrosis in the hearth ↑PGC1α and mitochondrial markers ↑utrophin protein ↓MMP9 protein expression ↑cardiac function ↑spontaneous activity	Ballmann et al. (2017a)
SIRT1 activation	8-week-old *mdx* mice fed 0.2% quercetin-enriched diet for 12 months	↓damage, inflammatory markers and fibrosis in the hearth ↑PGC1α and mitochondrial markers ↑antioxidant enzymes ↑spontaneous activity	Ballmann et al. (2017b)

Abbreviations: β-DG, β-dystroglycan; Bnip3, BCL2 Interacting Protein 3; Ca^2+^, calcium; CK, creatinkinase; COX, cytochrome c oxidase; CSA, cross-sectional area; DAPC, dystrophin-associated protein complex; EDL, extensor digitorum longus; FOXO3a, forkhead box O 3a; GAS, gastrocnemius; IP, intraperitoneal; LC3b, tubule-associated protein 1 light chain 3 beta; MMP-9, matrix metalloproteinase-9; MyHC, myosin heavy chain; NMJ, neuromuscular junction; nNOS, neuronal NO, synthase; NO, nitric oxide; NRF1, nuclear respiratory factor 1; OXPHOS, oxidative phosphorylation system; PGC-1α, peroxisome proliferator-activated receptor gamma coactivator 1-alpha; PPAR, peroxisome proliferator-activated receptor; PTP, permeability transition pore; RAGE, receptor for advanced glycation end-products; ROS, reactive oxygen species; RSV, resveratrol; Sgca: α-sarcoglycan; SIRT1, sirtuin1; SOD2, superoxide dismutase 2, mitochondrial; TA, tibialis anterior; TFEB, transcription factor EB; TGFβ1, transforming growth factor β1; TTP, time-to-peak; UCP-1, uncoupling Protein 1; Ulk1, unc-51-like kinase 1.

A number of pharmacological compounds that stimulate the activity of PPARδ, AMPK and SIRT1 have proven to be efficacious at relieving several DMD symptoms in animal models but there are several hurdles to overcome for their clinical development in DMD patients. The PPARδ selective agonist ASP0367 has shown a good safety profile in healthy voluntaries and has entered a clinical trial to test its effectiveness in boys with DMD, but unfortunately this study was terminated early due to enrollment challenges. The AMP analog AICAR has been the most used activator of AMPK in preclinical studies. However, AICAR exerts off-target effects and has shown a very poor oral bioavailability in clinical trials (Višnjić et al., 2021). Recently, more specific direct activators of AMPK have been identified and tested in preclinical studies, and few of them have already entered human clinical trials for type 2 diabetes and cardiovascular diseases (Steinberg and Carling, 2019). Metformin, a widely used anti-diabetic drug, also activates AMPK indirectly by inhibiting mitochondrial respiration. Initial clinical studies have tested the therapeutic effectiveness of a combined treatment of metformin and NO donors in DMD patients with encouraging results (Hafner et al., 2016a; Hafner et al., 2016b; Hafner et al., 2019). Furthermore, nutraceuticals such as the polyphenol resveratrol and the flavonoid quercetin, both endowed with antioxidant and anti-inflammatory properties, directly or indirectly activate SIRT1 and AMPK. RSV has been tested in many clinical trials on healthy adults of all ages and patients affected by various pathologies including metabolic syndrome, cancer, diabetes, and cardiovascular disorders. Overall, these clinical studies show that RSV was well-tolerated, consistently reduced inflammation and improved aspects of deregulated metabolism (Brown et al., 2024). A small open-label, single-arm pilot study has shown that RSV may provide some benefit to patients with Duchenne, Becker or Fukuyama muscular dystrophy (Kawamura et al., 2020). Clinical testing of quercetin has been carried out or is underway for multiple indications, showing some beneficial effects (Mirza et al., 2023). However, like other phytochemicals, RSV and quercetin present potential pharmaceutical challenges due to their high rate of metabolism and poor bioavailability. To overcome these limitations, more advanced oral formulations are under development.
